# Barriers and facilitators to uptake of non-surgical interventions for knee osteoarthritis: a protocol for a systematic review of qualitative studies

**DOI:** 10.3389/fmed.2026.1791068

**Published:** 2026-02-26

**Authors:** Ravi Shankar, Vahul Sundar, Matthew Rong Jie Tay

**Affiliations:** 1 Clinical Research and Innovation Office, Tan Tock Seng Hospital, National Healthcare Group, Singapore, Singapore; 2 Institute of Rehabilitation Excellence, Tan Tock Seng Hospital Rehabilitation Centre, Tan Tock Seng Hospital, Singapore, Singapore; 3 Yong Loo Lin School of Medicine, National University of Singapore, Singapore, Singapore; 4 Lee Kong Chian School of Medicine, Nanyang Technological University, Singapore, Singapore

**Keywords:** exercise therapy, implementation barriers and enablers, implementation science, knee osteoarthritis, non-surgical interventions, patient adherence, weight management

## Abstract

**Background:**

Knee osteoarthritis affects millions globally, causing pain, functional limitations, and reduced quality of life. Clinical guidelines recommend non-surgical interventions including exercise, weight management, and education as first-line treatments. However, uptake and adherence to these evidence-based interventions remain suboptimal, with many patients not receiving recommended care. While effectiveness evidence is robust, understanding of factors influencing implementation from patient and provider perspectives remains fragmented. Qualitative research provides rich insights into barriers and facilitators but lacks systematic synthesis to inform implementation strategies.

**Objectives:**

This systematic review protocol aims to synthesize qualitative evidence on barriers and facilitators to uptake of guideline-recommended non-surgical interventions for knee osteoarthritis, examining perspectives of patients, healthcare providers, and other stakeholders across diverse healthcare contexts.

**Methods:**

Following PRISMA-P and ENTREQ guidelines, we will search seven databases (MEDLINE, Embase, CINAHL, PsycINFO, AMED, Scopus, and Web of Science) from inception to December 2025. The PICo framework guides eligibility criteria focusing on adults with knee osteoarthritis, their experiences with non-surgical interventions, and contextual factors influencing uptake. Covidence will facilitate study screening and selection. Quality assessment will employ the Critical Appraisal Skills Program (CASP) qualitative checklist. Thematic synthesis following Thomas and Harden’s approach will identify descriptive and analytical themes. CERQual will assess confidence in synthesized findings.

**Discussion:**

This protocol establishes methodology for comprehensive synthesis of qualitative evidence on non-surgical intervention uptake for knee osteoarthritis. Findings will inform development of implementation strategies, clinical pathways, and patient support programs addressing identified barriers while leveraging facilitators. The synthesis will guide healthcare providers, policymakers, and researchers in improving delivery and uptake of evidence-based osteoarthritis care.

## Introduction

Knee osteoarthritis represents one of the most prevalent musculoskeletal conditions globally, affecting approximately 365 million people and ranking among the leading causes of disability worldwide (1). The condition is characterized by progressive joint degeneration involving cartilage loss, bone remodeling, osteophyte formation, and synovial inflammation, resulting in pain, stiffness, functional limitations, and reduced quality of life (2). With aging populations and rising obesity rates, knee osteoarthritis prevalence is projected to increase substantially, with estimates suggesting that nearly half of adults will develop symptomatic knee osteoarthritis during their lifetime (3). This growing burden places significant demands on healthcare systems, with osteoarthritis-related costs exceeding 100 billion dollars annually in the United States alone when considering direct medical expenses and indirect costs from lost productivity (4).

International clinical guidelines consistently recommend non-surgical interventions as first-line treatment for knee osteoarthritis, emphasizing core treatments that should be offered to all patients regardless of disease severity, comorbidities, or demographic characteristics (5). These core interventions include structured exercise programs encompassing strengthening, aerobic conditioning, and flexibility training; weight management strategies for overweight and obese individuals; and education about the condition, self-management strategies, and lifestyle modifications (6). Adjunct treatments such as manual therapy, assistive devices, and pharmacological options are recommended based on individual needs and preferences. The evidence supporting these interventions is robust, with systematic reviews demonstrating that exercise provides clinically meaningful improvements in pain and function comparable to pharmacological interventions but with fewer adverse effects (7). Weight loss of five to 10 % in overweight individuals can reduce pain by up to 50% and substantially improve physical function (8).

Despite strong evidence and consistent guideline recommendations, implementation of non-surgical interventions for knee osteoarthritis remains markedly suboptimal across healthcare systems globally. Studies indicate that fewer than 40% of patients with knee osteoarthritis receive advice about exercise, and less than 20% are referred to structured exercise programs (9). Weight management support is provided to fewer than half of overweight patients with knee osteoarthritis, with even lower rates of referral to comprehensive weight loss programs (10). This evidence-practice gap results in unnecessary suffering, functional decline, and premature progression to surgical interventions that might have been avoided or delayed with appropriate conservative management. Understanding why evidence-based non-surgical interventions are underutilized despite their proven effectiveness represents a critical challenge for improving osteoarthritis care.

The complexity of factors influencing uptake of non-surgical interventions for knee osteoarthritis extends across multiple levels. These range from individual patient beliefs and experiences, through healthcare provider attitudes and behaviors, to system-level constraints and societal influences. Patient-level factors include misconceptions about osteoarthritis as an inevitable consequence of aging requiring rest rather than activity, fear of movement and exercise causing further joint damage, previous negative experiences with conservative treatments, and expectations that only surgical or pharmacological interventions provide meaningful relief (11). Healthcare provider factors encompass nihilistic attitudes about osteoarthritis treatment possibilities, limited knowledge about evidence-based interventions, time constraints preventing comprehensive patient education, and uncertainty about referral pathways for exercise and weight management programs (12). System factors include limited access to appropriate services, cost barriers, long waiting times, and fragmentation between primary and specialist care.

Qualitative research methods provide unique insights into these complex, multifaceted barriers and facilitators by exploring lived experiences, meanings, and contexts that quantitative approaches cannot fully capture. Qualitative studies have examined patient perspectives on exercise for knee osteoarthritis, revealing complex belief systems about activity and rest, social influences on exercise participation, and emotional responses to changing physical capabilities (13). Provider perspectives have been explored through qualitative research identifying professional role boundaries, confidence in delivering interventions, and perceived patient receptiveness as key influences on treatment recommendations (14). However, this rich qualitative evidence remains fragmented across individual studies conducted in diverse contexts with varying populations and healthcare systems. Systematic synthesis is needed to identify patterns across studies, develop comprehensive understanding of implementation challenges, and generate actionable insights for improving intervention uptake.

This systematic review aims to synthesize qualitative evidence on barriers and facilitators to uptake of guideline-recommended non-surgical interventions for knee osteoarthritis. The primary objective is to identify and characterize factors influencing patient engagement with core non-surgical treatments including exercise, weight management, and education across diverse populations and healthcare contexts. The review will examine perspectives of multiple stakeholders including patients with knee osteoarthritis, healthcare providers delivering care, and other relevant parties such as family members and healthcare administrators to develop comprehensive understanding of implementation influences.

The review addresses several research questions central to understanding non-surgical intervention uptake. The primary research question asks: What are the barriers and facilitators to uptake of guideline-recommended non-surgical interventions for knee osteoarthritis from the perspectives of patients and healthcare providers? Secondary questions explore: How do barriers and facilitators vary across different non-surgical interventions such as exercise versus weight management? What are the similarities and differences in perspectives between patients and healthcare providers regarding intervention uptake? How do contextual factors including healthcare system characteristics, cultural backgrounds, and socioeconomic circumstances influence barriers and facilitators? What strategies have been identified for overcoming barriers and enhancing facilitators to improve intervention uptake?

These questions recognize that intervention uptake is influenced by complex interactions between individual, interpersonal, organizational, and societal factors. Such complexity requires comprehensive examination across multiple dimensions.

## Methods

### Study design and protocol registration

This qualitative systematic review protocol follows the Preferred Reporting Items for Systematic Reviews and Meta-Analyses Protocols (PRISMA-P) 2015 guidelines ensuring transparent reporting of planned methods (15). The protocol will be registered with the International Prospective Register of Systematic Reviews (PROSPERO) before commencing searches to promote transparency and reduce duplication. The review will employ the Enhancing Transparency in Reporting the Synthesis of Qualitative Research (ENTREQ) statement for reporting qualitative synthesis and the Joanna Briggs Institute methodology for systematic reviews of qualitative evidence (16, 17). Any protocol amendments will be documented with dates, descriptions, and rationales. The review team includes expertise in osteoarthritis management, qualitative research methods, implementation science, and systematic review methodology, ensuring appropriate perspectives for comprehensive synthesis.

The review team comprises three members. RS will lead the search strategy, screening, data extraction, and synthesis. VS will serve as the second independent reviewer for title/abstract and full-text screening, data extraction, and quality appraisal. MRT will provide clinical expertise in osteoarthritis management, act as the third reviewer for resolving disagreements, and oversee methodological decisions throughout the review process.

### Conceptual framework

The PICo framework (Population, phenomenon of Interest, Context) structures this systematic review, providing appropriate organization for qualitative evidence synthesis (18). The Population encompasses adults with knee osteoarthritis of any severity, duration, or etiology, including those with isolated knee osteoarthritis and those with multi-joint involvement where knee-specific data can be extracted. Healthcare providers involved in knee osteoarthritis management including general practitioners, rheumatologists, orthopedic surgeons, physiotherapists, occupational therapists, nurses, and other relevant professionals are also included. Family members, caregivers, and other stakeholders are eligible when their perspectives relate to patient uptake of interventions.

The phenomenon of Interest captures barriers and facilitators to uptake of guideline-recommended non-surgical interventions for knee osteoarthritis. This includes factors influencing initial engagement, ongoing participation, and sustained adherence to interventions. Core interventions of interest comprise exercise in any format including land-based, aquatic, supervised, or home programs; weight management encompassing dietary modification, physical activity for weight loss, and behavioral interventions; and education about osteoarthritis, self-management, and lifestyle modification. Adjunct interventions such as manual therapy, assistive devices, and complementary therapies are included when examined alongside core treatments. The review focuses on factors influencing real-world implementation rather than efficacy under controlled conditions.

The context component acknowledges that intervention uptake occurs within complex healthcare and social environments. All healthcare settings are eligible including primary care, specialist clinics, hospitals, community facilities, and home-based care. Studies from all countries and healthcare systems are included without language restrictions, recognizing that cultural, economic, and organizational factors profoundly influence intervention implementation. The temporal context spans from initial diagnosis through ongoing management, acknowledging that barriers and facilitators may evolve across the osteoarthritis trajectory.

While no single theoretical framework is imposed *a priori* on the synthesis, findings may be mapped *post hoc* to established implementation science frameworks such as the Theoretical Domains Framework (TDF) or the Capability, Opportunity, Motivation–Behavior (COM-B) model (19, 20). This approach preserves the inductive nature of thematic synthesis while enabling structured interpretation that can inform theory-driven implementation strategies.

### Eligibility criteria

Population criteria include adults aged 18 years and older with knee osteoarthritis diagnosed through clinical criteria, radiographic evidence, or self-report consistent with osteoarthritis symptoms. Studies including mixed arthritis populations are eligible if knee osteoarthritis data can be extracted separately. Healthcare providers of any profession involved in delivering non-surgical interventions for knee osteoarthritis are included regardless of experience level or practice setting. Studies examining perspectives of family members, informal caregivers, or healthcare administrators are eligible when focused on factors influencing patient intervention uptake.

The phenomenon of interest encompasses experiences, perceptions, attitudes, and beliefs about barriers and facilitators to engaging with non-surgical interventions for knee osteoarthritis. Studies must explicitly examine factors influencing intervention uptake, adoption, participation, or adherence rather than solely examining intervention effectiveness or satisfaction. Research exploring decision-making processes, motivation, behavior change, and implementation experiences are included. Studies focused exclusively on surgical interventions, pharmacological treatments without non-surgical components, or diagnostic procedures are excluded.

Study design criteria require qualitative methodology providing rich descriptive data about barriers and facilitators. Eligible designs include phenomenology exploring lived experiences of intervention engagement, grounded theory developing theoretical understanding of uptake processes, ethnography examining cultural influences on intervention participation, descriptive qualitative studies identifying barriers and facilitators, and action research documenting implementation experiences. Mixed-methods studies are eligible if qualitative components can be extracted and meet inclusion criteria. For mixed-methods studies, the qualitative component will be identified based on the presence of distinct qualitative data collection methods (e.g., interviews, focus groups, open-ended survey responses) and qualitative analytical approaches (e.g., thematic analysis, content analysis, grounded theory). Quality appraisal using CASP will focus solely on the qualitative component of such studies, assessing the rigour of qualitative data collection, analysis, and reporting independently of the quantitative elements. Studies in which qualitative findings are limited to brief open-ended responses without systematic qualitative analysis, or where qualitative data serve only as supplementary illustration of quantitative findings without independent qualitative inquiry, will be excluded. Purely quantitative studies, intervention effectiveness trials without qualitative process evaluation, and opinion pieces without empirical data are excluded (Table 1).

**Table 1:** Inclusion and exclusion criteria.

Criteria	Inclusion	Exclusion
Population	Adults (≥18 years) with knee osteoarthritis; Healthcare providers managing knee osteoarthritis; Family/caregivers influencing uptake	Children/adolescents; Other arthritis types without knee osteoarthritis data
Phenomenon	Barriers and facilitators to non-surgical intervention uptake; Implementation experiences; Adherence factors	Intervention effectiveness only; Exclusively surgical or pharmacological focus
Context	All healthcare settings; All countries; Any stage of osteoarthritis management	Non-healthcare contexts
Study Design	Qualitative studies with primary data; Mixed-methods with extractable qualitative component	Quantitative only; Reviews; Opinion pieces
Intervention	Exercise, weight management, education, self-management, adjunct therapies	Surgical interventions only; Diagnostics only
Time Period	Database inception to date of final search (initial search planned March 2026)	None

### Information sources and search strategy

Comprehensive database searching will encompass seven major databases covering health, psychology, and rehabilitation literature. MEDLINE via PubMed will capture biomedical literature including clinical and health services research. Embase will provide international coverage including European and Asian studies often not indexed elsewhere. CINAHL will identify nursing and allied health perspectives crucial for understanding multidisciplinary intervention delivery. PsycINFO will capture psychological and behavioral aspects of intervention engagement. AMED (Allied and Complementary Medicine Database) will include rehabilitation and complementary therapy perspectives. Scopus and Web of Science will provide broad interdisciplinary coverage and enable citation tracking.

The search strategy combines four concept blocks refined through consultation with an information specialist experienced in qualitative systematic reviews. Knee osteoarthritis terms include “knee osteoarthritis,” “knee osteoarthrosis,” “knee arthritis,” “knee degeneration,” and “gonarthrosis.” Non-surgical intervention terms encompass “exercise,” “physical activity,” “weight management,” “diet,” “education,” “self-management,” “conservative treatment,” and “non-pharmacological.” Barriers and facilitators terms include “barriers,” “facilitators,” “enablers,” “obstacles,” “challenges,” “uptake,” “adherence,” “compliance,” “implementation,” and “engagement.” Qualitative research terms comprise “qualitative,” “interview,” “focus group,” “ethnography,” “phenomenology,” “grounded theory,” “thematic analysis,” and “content analysis.” The strategy accommodates terminology variations across disciplines and publication periods while maintaining sensitivity for qualitative research identification. The initial database searches are planned for March 2026, with an updated search to be conducted immediately prior to final analysis to capture any newly published studies. The date of each search will be recorded and reported in accordance with PRISMA-P guidance.

Supplementary search strategies will identify additional relevant studies through multiple approaches. Reference lists of included studies and relevant systematic reviews will be hand-searched. Forward citation searching using Google Scholar and Web of Science will identify studies citing key papers. Gray literature searching will include ProQuest Dissertations and Theses Global, conference proceedings from osteoarthritis and rehabilitation conferences, and websites of arthritis organizations and research centers. Content experts in osteoarthritis management and qualitative research will be contacted to identify unpublished or in-press studies. Authors of conference abstracts will be contacted for full study details where available.

### Study selection

Covidence systematic review software will manage the screening process, providing systematic tracking of decisions and reviewer agreement (21). Two reviewers will independently screen titles and abstracts against basic eligibility criteria, followed by full-text assessment of potentially eligible studies. Before commencing screening, reviewers will complete calibration exercises using 50 randomly selected citations to ensure consistent application of criteria. Inter-rater reliability will be calculated using Cohen’s kappa statistics with values above 0.75 indicating acceptable agreement.

The screening process involves initial assessment of titles and abstracts for basic eligibility focusing on population, phenomenon, and study design. Studies marked as potentially eligible by either reviewer proceed to full-text assessment. Full-text screening examines all eligibility criteria with specific reasons for exclusion documented. Disagreements between reviewers are resolved through discussion, with a third reviewer consulted when consensus cannot be reached. Authors are contacted when additional information is needed to determine eligibility, with three contact attempts over 4 weeks before exclusion. The PRISMA flow diagram will document study flow through screening stages including reasons for full-text exclusions.

### Data extraction

A comprehensive data extraction form developed specifically for qualitative evidence will capture study characteristics, methodological details, and findings relevant to barriers and facilitators. The form was developed based on JBI guidance for qualitative data extraction and piloted on five studies to ensure completeness and usability. Two reviewers will independently extract data from each included study with discrepancies resolved through discussion and return to original sources. Authors will be contacted to clarify unclear information or obtain additional details about methods or findings.

Study characteristics extracted include author information, publication year, country and setting, funding sources, and declared conflicts of interest. Population characteristics encompass sample size and composition, participant demographics including age, gender, disease duration and severity, recruitment methods and settings, and inclusion and exclusion criteria. For healthcare provider participants, professional backgrounds, experience levels, and practice settings will be recorded. Methodological details include qualitative approach and theoretical framework, data collection methods and procedures, data analysis techniques, strategies for ensuring rigor and trustworthiness, and reflexivity statements about researcher positioning.

Intervention characteristics will document which non-surgical interventions were examined, how interventions were delivered in local contexts, and any implementation strategies employed. Findings extraction will focus on identified barriers organized by levels such as patient, provider, and system factors; identified facilitators similarly organized; participant quotations illustrating key barriers and facilitators; authors’ interpretations and theoretical developments; and any proposed strategies for improving uptake. Contextual information about healthcare system characteristics, cultural factors, and local implementation conditions will be extracted to enable assessment of finding transferability.

### Quality assessment

The Critical Appraisal Skills Program (CASP) Qualitative Research Checklist will assess methodological quality of included studies (22). This tool examines 10 key areas including clarity of research aims, appropriateness of qualitative methodology, research design suitability for addressing aims, recruitment strategy appropriateness, data collection methods addressing research issues, consideration of researcher-participant relationships, ethical considerations, rigor of data analysis, clarity of findings presentation, and research value. Two reviewers will independently assess each study with disagreements resolved through discussion.

Quality assessment will inform confidence in individual study contributions to synthesis findings rather than determining study exclusion. This approach recognizes that even studies with methodological limitations may provide valuable insights about barriers and facilitators. Studies will be categorized as high quality when meeting 8–10 CASP criteria, moderate quality when meeting five to seven criteria, or low quality when meeting fewer than five criteria. Gray literature sources, including dissertations, theses, and conference proceedings with sufficient methodological detail, will be appraised using the same CASP checklist applied to published studies. Where gray literature sources lack complete reporting of methodological procedures, this will be reflected in lower CASP ratings. In CERQual assessments, gray literature will not be weighted differently from published studies per se; however, methodological limitations identified through CASP appraisal will be factored into the methodological limitations component of CERQual, and any concerns about the adequacy or transparency of reporting will be documented. This approach ensures that gray literature can contribute valuable insights while maintaining rigorous quality standards. Sensitivity analyses will examine whether synthesis findings differ when lower quality studies are excluded. Temporal trends in study quality will be examined to understand methodological evolution in this field.

### Data synthesis

Thematic synthesis following Thomas and Harden’s three-stage approach will integrate findings across studies while preserving interpretive richness (23). This method was selected for its systematic approach to identifying patterns across studies while enabling development of new interpretive insights beyond individual study findings. NVivo qualitative analysis software will facilitate coding and theme development while maintaining an audit trail of analytical decisions.

The first stage involves line-by-line coding of study findings sections including participant quotations and author interpretations. Initial codes will remain close to the original data using participants’ own words where possible. Two reviewers will independently code five studies, comparing coding to develop a preliminary framework that will be refined throughout the synthesis. The second stage organizes codes into descriptive themes capturing patterns across studies. Descriptive themes will be developed separately for barriers and facilitators, with attention to different stakeholder perspectives and intervention types.

Barriers and facilitators related to adjunct interventions will not be synthesized as standalone themes. Rather, where studies report findings on adjunct interventions alongside core treatments, these data will be integrated into the synthesis only to the extent that they illuminate factors influencing uptake of core interventions (exercise, weight management, and education). Findings pertaining exclusively to adjunct interventions without relevance to core intervention uptake will be treated as contextual information and reported descriptively rather than incorporated into the thematic synthesis.

Constant comparison will identify similarities and differences across contexts and populations. The third stage develops analytical themes that go beyond individual study findings to generate new interpretive insights about factors influencing intervention uptake. These analytical themes will explore relationships between barriers and facilitators, mechanisms through which factors operate, and conditions under which factors become more or less influential.

Subgroup analyses will explore whether barriers and facilitators differ across key dimensions. Intervention-specific analyses will compare factors influencing exercise uptake versus weight management versus education, identifying common and unique challenges. Stakeholder comparisons will examine alignment and divergence between patient and provider perspectives. Contextual analyses will explore how healthcare system types, cultural backgrounds, and socioeconomic factors shape barriers and facilitators. Disease-related analyses will examine whether factors differ based on osteoarthritis severity, duration, or presence of comorbidities. These analyses will identify whether certain barriers and facilitators are universal versus context-specific.

### Confidence assessment

The Confidence in Evidence from Reviews of Qualitative research (CERQual) approach will assess confidence in synthesized findings (24). This framework evaluates four components that may affect confidence in qualitative synthesis findings. Methodological limitations assess the extent to which studies contributing to a finding have methodological problems based on CASP assessments. Coherence evaluates whether findings are well-grounded in data from contributing studies and provide convincing explanations. Adequacy of data examines whether studies provide sufficiently rich data and whether enough studies contribute to develop understanding. Relevance assesses the extent to which contributing studies are applicable to the review question’s context, population, and phenomenon.

Each synthesized finding will receive an overall CERQual confidence rating of high, moderate, low, or very low based on assessment across all four components. Two reviewers will independently conduct CERQual assessments with disagreements resolved through discussion. Summary of Qualitative Findings tables will present each finding with its confidence assessment and explanation of the judgment. This transparent presentation enables users to understand the strength of evidence supporting each finding and make informed decisions about applicability to their contexts.

## Discussion

This systematic review protocol establishes rigorous methodology for synthesizing qualitative evidence on barriers and facilitators to non-surgical intervention uptake for knee osteoarthritis. The synthesis addresses a critical implementation gap where effective interventions proven to reduce pain and improve function remain underutilized in clinical practice. By systematically examining factors influencing intervention uptake from multiple stakeholder perspectives, the review will generate comprehensive understanding essential for developing targeted implementation strategies. The protocol’s strength lies in its focused scope on implementation factors rather than intervention effectiveness. Evidence of what works must be complemented by understanding of how to ensure interventions reach those who need them.

The review occurs at an important juncture in osteoarthritis care, as growing evidence challenges traditional biomedical models and emphasizes active management through exercise and lifestyle modification (25, 26). However, translating this paradigm shift into practice requires addressing deeply embedded beliefs among patients and providers, restructuring service delivery models, and ensuring equitable access. The qualitative synthesis will illuminate these complex implementation challenges that quantitative effectiveness research cannot fully address.

The PICo framework provides appropriate structure for this qualitative synthesis, accommodating the exploratory nature of barrier and facilitator identification while maintaining systematic rigor. Unlike frameworks designed for intervention effectiveness questions, PICo recognizes that qualitative research explores phenomena within contexts rather than measuring outcomes of specific interventions. This alignment with qualitative research purposes ensures comprehensive capture of relevant studies examining implementation experiences across diverse settings and populations. The framework’s flexibility enables inclusion of various stakeholder perspectives and intervention types while maintaining clear boundaries for the review scope.

Several anticipated challenges require consideration during review conduct. Heterogeneity in how barriers and facilitators are conceptualized and reported across studies may complicate synthesis. Some studies may use theoretical frameworks such as the Theoretical Domains Framework or COM-B model, while others employ inductive approaches without predetermined categories. The synthesis will accommodate this diversity by mapping findings to common conceptual categories while preserving unique insights from different theoretical perspectives. This approach balances comprehensive integration with respect for diverse analytical approaches contributing different understanding dimensions.

Cultural and contextual variation in factors influencing intervention uptake presents both challenges and opportunities for the synthesis. Barriers related to healthcare access and cost may predominate in some settings, while cultural beliefs about aging and activity may be more influential elsewhere. The synthesis will explore these variations through subgroup analyses while identifying universal themes transcending specific contexts. Understanding both common and context-specific factors enables development of implementation strategies that address core challenges while allowing local adaptation. This balance between generalizability and contextual sensitivity maximizes the review’s utility for diverse audiences globally.

The review’s findings will have immediate practical applications for improving osteoarthritis care delivery. For healthcare providers, understanding patient-perceived barriers enables more effective communication addressing specific concerns rather than generic information provision. Recognition of system-level barriers can guide advocacy for service improvements and resource allocation. For healthcare organizations, identified barriers and facilitators inform service design decisions such as program location, timing, and delivery format. Understanding facilitators enables services to build on existing strengths and motivations rather than focusing solely on deficit correction.

Implementation strategies can be tailored based on predominant barriers identified in specific contexts. If misconceptions about exercise causing joint damage emerge as major barriers, educational interventions addressing these beliefs become priorities. If access and convenience are primary obstacles, alternative delivery models such as telehealth or community-based programs may be solutions. The synthesis will enable evidence-based selection of implementation strategies matched to identified barriers rather than generic approaches that may miss key obstacles. This targeted approach increases likelihood of successful implementation and sustained intervention uptake.

The review will contribute to theoretical understanding of health behavior change in chronic condition management. Examining barriers and facilitators through multiple theoretical lenses will identify which frameworks best explain intervention uptake in osteoarthritis contexts. The synthesis may reveal that certain theories better account for exercise engagement while others better explain weight management participation. Understanding theoretical mechanisms through which barriers operate and facilitators function enables development of theory-informed implementation strategies with clear logic models linking strategies to outcomes.

Several limitations merit acknowledgment to frame appropriate interpretation of findings. The review focuses on qualitative evidence providing deep understanding of barriers and facilitators but cannot quantify their relative importance or prevalence. Complementary quantitative research would be needed to determine which barriers affect the most people or have greatest impact on intervention uptake. The synthesis cannot establish causal relationships between identified factors and actual intervention participation, as most qualitative studies explore perceptions rather than measuring behaviors. However, understanding perceived barriers remains crucial as perceptions shape behavior regardless of objective accuracy.

The broad inclusion criteria encompassing diverse populations, interventions, and contexts may limit depth of analysis for specific subgroups or interventions. Subsequent focused reviews might examine specific populations such as older adults or specific interventions such as aquatic exercise in greater detail. Language restrictions were not applied, but practical limitations may affect inclusion of studies in languages beyond team capabilities. Professional translation will be sought for potentially eligible studies, but some relevant evidence may be missed. The focus on published studies may exclude innovative implementation approaches not yet formally evaluated, though gray literature searching partially addresses this limitation.

In summary, the protocol outlines a systematic methodology for synthesizing qualitative evidence on a critical implementation challenge in osteoarthritis care. The comprehensive approach ensures identification and integration of diverse perspectives on barriers and facilitators while maintaining quality standards appropriate for qualitative research. Findings will provide essential evidence for developing implementation strategies that address real-world obstacles to intervention uptake rather than assuming that evidence of effectiveness automatically translates to practice change. The synthesis contributes to broader implementation science by demonstrating how qualitative evidence synthesis can illuminate complex implementation challenges and inform targeted strategies for evidence-based practice adoption. Ultimately, understanding and addressing barriers while leveraging facilitators to non-surgical intervention uptake can improve care quality and outcomes for millions living with knee osteoarthritis globally.
